# Mental disorders and the use of primary health care services among homeless shelter users in the Helsinki metropolitan area, Finland

**DOI:** 10.1186/s12913-017-2372-3

**Published:** 2017-06-21

**Authors:** Agnes Stenius-Ayoade, Peija Haaramo, Elisabet Erkkilä, Niko Marola, Kirsi Nousiainen, Kristian Wahlbeck, Johan G. Eriksson

**Affiliations:** 1 Folkhälsan Research Center, Helsinki, Finland; 2 National Institute for Health and Welfare, Mental Health Unit, Helsinki, Finland; 3 Department of Social Services and Health Care, City of Helsinki, Helsinki, Finland; 4 Department of Social Research, University of Helsinki, Helsinki, Finland; 5 The Finnish Association for Mental Health, Helsinki, Finland; 6 National Institute for Health and Welfare, Department of Chronic Disease Prevention, Helsinki, Finland; 7 Department of General Practice and Primary Health Care, University of Helsinki, Helsinki, Finland

**Keywords:** Homeless, Primary health care use, Mental disorders, Register based cohort study, Shelter use, Dual diagnosis

## Abstract

**Background:**

Homelessness is associated with increased morbidity, mortality and health care use. The aim of this study was to examine the role of mental disorders in relation to the use of 1) daytime primary health care services and 2) after hours primary health care emergency room (PHER) services among homeless shelter users in the Helsinki Metropolitan Area, Finland.

**Methods:**

The study cohort consists of all 158 homeless persons using the four shelters operating in the study area during two selected nights. The health records were analyzed over a period of 3 years prior to the sample nights and data on morbidity and primary health care visits were gathered. We used negative binomial regression to estimate the association between mental disorders and daytime visits to primary health care and after hours visits to PHERs.

**Results:**

During the 3 years the 158 homeless persons in the cohort made 1410 visits to a physician in primary health care. The cohort exhibited high rates of mental disorders, including substance use disorders (SUDs); i.e. 141 persons (89%) had a mental disorder. We found dual diagnosis, defined as SUD concurring with other mental disorder, to be strongly associated with daytime primary health care utilization (IRR 11.0, 95% CI 5.9–20.6) when compared with those without any mental disorder diagnosis. The association was somewhat weaker for those with only SUDs (IRR 4.9, 95% CI 2.5–9.9) or with only other mental disorders (IRR 5.0, 95% CI 2.4–10.8). When focusing upon the after hours visits to PHERs we observed that both dual diagnosis (IRR 14.1, 95% CI 6.3–31.2) and SUDs (11.5, 95% CI 5.7–23.3) were strongly associated with utilization of PHERs compared to those without any mental disorder. In spite of a high numbers of visits, we found undertreatment of chronic conditions such as hypertension and diabetes.

**Conclusions:**

Dual diagnosis is particularly strongly associated with primary health care daytime visits among homeless persons staying in shelters, while after hours visits to primary health care level emergency rooms are strongly associated with both dual diagnosis and SUDs. Active treatment for SUDs could reduce the amount of emergency visits made by homeless shelter users.

## Background

Homeless people constitute a highly vulnerable part of the population with multiple social and health care needs [1, 2]. Homelessness has been found to be associated with increased morbidity and mortality in several settings [1, 3–10]. A systematic review on mental health disorders among homeless persons in Western countries by Fazel and coworkers showed that the most common mental disorders among homeless persons were alcohol dependence and drug dependence followed by psychotic illness and major depression [6]. Dual diagnosis with concurrent substance use disorders (SUDs) and other mental disorders is also commonly presenting among homeless persons using shelters and care programs for homeless [4, 5, 11, 12].

Additionally, the somatic disease burden has been shown to be high among homeless persons, with increased rates of infectious diseases, such as human immunodeficiency virus (HIV), hepatitis, tuberculosis and pneumonia; and chronic medical conditions, such as cardiovascular disease, obesity and chronic obstructive lung disease [1]. Both somatic and mental illnesses are predictors of mortality among homeless persons [13, 14]. Swedish and Danish studies have shown that SUDs and dual diagnosis especially are associated with high mortality among homeless persons [3, 5].

Studies from several settings have shown that homeless persons have more hospitalizations and emergency department (ED) visits than the general population [1, 8, 12, 15–17]. However, utilization of primary health care services seems to differ from one setting to another. In the United States, a setting with an insurance based health care system, the utilization of ambulatory health care for homeless persons using missions shelters is lower than that for the general population [18] and homeless persons use EDs for their primary health care needs [19]. While in Canada and Belgium, settings with universal health care and therefore lower barriers to primary health care, homeless persons using shelters and meal programs do use ambulatory services and primary health care more than the general population, though the use of EDs and hospital services still remain high [20, 21]. We know that the homeless persons use ED for visits related to injuries, substance use and psychiatric conditions [22–24] but to our knowledge, there are no previous studies on the reasons why homeless persons seek primary health care services.

Several barriers to care have been identified such as stigmatizing attitudes of health care personnel, lack of insurance, or difficulties registering with general practitioners, as well as competing more immediate needs such as food and shelter [25, 26]. To meet the primary health care needs of the homeless population many bigger cities have developed systems with targeted primary health care for homeless and some studies suggest that these are more efficient in reaching the homeless population [27, 28]. There is suggestive evidence that tailored primary health care services for homeless persons improve the delivery of care at the right level and reduce the number of nonacute ED visits [29, 30].

Health service use has been shown to be unevenly distributed in the homeless population, with a small proportion of the homeless persons being responsible for a majority of hospitalizations and ED visits [1, 20, 31]. Chronic health conditions and mental health and substance use problems are factors that are known to be associated with ED use among homeless persons [12, 31–33]. However, we still lack knowledge about how mental disorders and chronic health conditions are associated with the use of primary health care services in the homeless population.

In November 2008, at the time of the sampling for this study, there were 7955 single homeless persons in Finland and in addition to them about 300 homeless families [34]. These national statistics on homelessness in Finland are gathered yearly by the Housing Finance and Development Centre of Finland (ARA) as cross-sectional data and despite their apparent exactness they are estimates made with varying methods in different municipalities. Out of Finland’s 7955 single homeless persons 4247 (53%) lived in the capital region [34]. The gender distribution of the homeless people in Finland was 80% male and 20% female [34]. The definition of homeless in Finland includes persons staying with families or friends and persons in institutions such as hospitals or prisons but without a permanent address. Due to the hard Finnish climate only a very few homeless persons actually sleep rough, and in the national statistics those sleeping outdoors are reported grouped together with those staying in shelters (162 persons in Helsinki, Vantaa and Espoo in 2008 were sleeping rough or in shelters [34]).

The aim of this study was to examine the prevalence of mental disorders in the shelter population in Helsinki metropolitan area, Finland, and to analyze how much these homeless persons seek primary health care services daytime and during after hours. We also wanted to study whether there is a relationship between the mental disorders and service utilization. We chose to focus on the homeless staying in shelters since these individuals, with the exception of those few individuals sleeping outdoors, constitute the most vulnerable group in the homeless population and could be sampled using the registers from the shelters.

## Methods

The study cohort consists of all 158 homeless persons using the four shelters operating in the Helsinki metropolitan area during two selected nights (16th of June 2008 and 16th of September 2008). The nights were selected 3 months apart in order to catch more of the short-term homeless population who spent only a shorter period in shelters. The selected nights were a Monday and a Tuesday (in Helsinki metropolitan area the level of shelter usage is not higher during particular days of the week). For those 45 homeless persons who used the shelters on both of the selected nights the latter one was used as the sample date. The shelter services in Helsinki metropolitan area are free of charge and open to all homeless residents on a walk-in basis, but as the services are not automatically available for non-local residents, homeless persons without place of domicile such as unregistered migrants and tourists without lodging do not have access to shelter services and are therefore not included in this study.

### Data collection

Materials used in this study consist of electronic health records made by physicians and nurses working in primary health care in the three municipalities. These data were retrieved from the electronic health care record databases of the respective municipalities. The health records and the textual content in the visits were analyzed for a period of 3 years prior to the sample night. We used personal identification codes that are unique to all citizens in Finland to link the study sample with the electronic health records in each of the municipalities. Immediately after retrieving the health records, they were anonymized by removing personal identification numbers and names and replacing them with study identification numbers. Analyses were made without these identification details. The linking and analyses were performed by a physician (first author). Electronic health records in the most recent register were introduced in 2004, hampering the possibility of a longer analysis period in this study. We analyzed all such visits to primary health care where the patient met a physician. Only the visits during which the patient met a physician face to face were analyzed, while phone calls, no-shows, and visits to nurses or other health care professionals were not included. If the patient had been examined by several physicians during one visit it was counted as one visit unless the patient was transferred from one unit to the other, for example from the health care center to primary health care emergency room in which case it was counted as two visits.

We analyzed the textual content of each visit and considered all diseases mentioned in the health records during the observation period. Only diseases mentioned by physicians were counted as diagnoses, suspicions or diseases mentioned by other professionals were not included. The diseases mentioned in the text were classified according to the ICD-10 classification system [35]. To calculate the prevalence of SUDs we counted obvious references to a substance use such as “alcoholic” or “history of problematic drug use” or “a professional alcoholic”, but subtle references such as “smelling of alcohol” or “patient denies problems with alcohol” were not included. Patients who had been referred to detox treatment or who had been in “sobering-up unit” because of intoxication in the analyzed period were also considered to have a diagnosis of SUDs.

Primary health care in Finland is organized by the municipalities, who are responsible for providing health care for all residents. In 2008, the municipalities of the Helsinki metropolitan area (approximately 850,000 inhabitants) provided primary health care services in 43 daytime primary health centers. The municipalities also organize primary health care level after hours emergency room (PHER) services in conjunction with hospitals or health centers. These PHERs handle simpler medical emergencies and conditions treatable by generalists, such as minor traumas and infections. In 2008 there were six PHERs in the area. In this study we analyzed all visits made by the persons belonging to the sample to primary health care centers day time and to PHERs during the study period. We did not have access to visits made based on direct referral to specialized care, mainly provided by the Hospital district in Helsinki and Uusimaa area (HUS). The City of Helsinki, however, also provides specialist care in internal medicine and psychiatry and since these hospitals use the same electronic health record system as primary health care, their health records were also used to gather data on morbidity of the homeless persons from the city of Helsinki (*N* = 89). The background demographic data of the study sample such as the socioeconomic status, housing history and history of treatment for SUDs were gathered by a social worker from the social service client registers. The register contains data on all housing services and in-patient detox treatment financed by the municipality as well as client notes taken by social workers. These data, however, were available to us only on the level of the whole cohort and thus could not be linked to the health care data on an individual level.

### Variables used in the study

Our outcome was primary health care service use, defined as the number of visits to primary health care centers during daytime and number of visits to PHER after hours.

The visits were grouped for the analyses according to place of healthcare delivery (i.e. primary health center or PHER) and the main reasons for seeking help. Though the official recommendation is to always enter a diagnostic code for each visit, this is far from always done; therefore, in determining the main reason for seeking help we also analyzed the textual content of the visit note. To form classifications for reasons for seeking help, 10% of the samples’ visits were analyzed and the commonly occurring main reasons were identified. These groups were 1) mental health and substance related problems, 2) traumas (e.g. wounds, fractures, concussions, strains, and physical abuse), 3) infections, 4) intoxications and convulsions, 5) diseases of the musculoskeletal system, 6) diseases of the gastrointestinal system, and 7) other reasons. Visits with more than one equally important reason, visits for which the main reason remained unclear, and visits to emergency room due to lack of shelter were also included in the group “other reasons”.

Whether a person was classified as having a mental disorder was based on the presence of a psychiatric diagnosis in the health record diagnosis field or textual documentation. We identified and grouped the sample into persons with 1) no mental disorder, 2) SUDs 3) mental disorder other than SUDs and 4) dual diagnosis, persons with both SUDs and other mental disorder other than SUDs. SUDs (group 2) included mental and behavioral disorders due to use of alcohol, benzodiazepines or illegal drugs. Mental disorders other than SUDs (group 3) included schizophrenia and other psychoses, bipolar disorder, depression, anxiety disorders, and other mental disorders.

We also assessed whether somatic comorbidity was related to the amount of visits to primary health care during daytime or visits to the PHER after hours. Prognostic comorbidity was calculated using the Charlson comorbidity index, originally created to help predict long term mortality [36]. The Charlson comorbidity index has been found reliable and valid in predicting mortality and length of hospitalization [37, 38]. The index is a list of 17 conditions, with each condition assigned a weight of 1, 2, 3, or 6, where HIV and metastatic carcinoma give the highest weight of 6 and less severe conditions such as mild liver disease and diabetes without complications give a score of 1. The noted diagnoses in the primary health care records during the analyzed period were converted into ICD-10 codes and used to calculate the Charlson comorbidity index in the study population.

### Statistical methods

To account for over-dispersion in the outcome variables we used the negative binomial regression to estimate the association between the primary independent variable (mental disorder) and the outcome variables (daytime visits to primary health care and after hours visits to PHERs). The assumptions of over-dispersion in the Poisson model were tested using Lagrange multiplier test. We also tested possible multicolinearity using variance inflation factors (VIF). The results are shown as incident rate ratios (IRRs) with their 95% confidence intervals. The IRRs represent the ratio of the count of visits for the variable of interest (mental disorder) to its reference group (people with no mental disorder). We examined three different models. Model 1 is a crude model, in Model 2 we adjusted for age and gender, and in Model 3 we controlled additionally for comorbidity using Charlson comorbidity index score as a continuous variable. Statistical comparison of main reasons for visits between primary health centers and PHER was performed using bootstrap-type t-test (5000 replications), since the data were highly skewed. The data analysis was done using IBM SPSS Statistics for Windows, version 23 (IBM Corp., Armonk, N.Y., USA).

## Results

The study sample included 158 homeless persons. Their mean age was 47.3 years (range 18–73), they were predominantly men (73.4%), Finnish citizens (95.6%) and unemployed (43.7%) or living on age-related or disability pensions (43.7%) (Table 1). Many of the study subjects had been homeless for a long time, more than half (55.1%) had been homeless for at least 1 year and almost a quarter (23.4%) for more than 5 years. Repeated previous periods of homelessness were also observed in more than a third of the sample. There was a large variance in the length of stay in shelter, some had stayed only for a few days, while others had lived in the shelter for many years (71.5% had stayed for more than a month, 7.6% over 11 months during the last year).

**Table 1:** Characteristics of the study sample, homeless persons staying in shelters in Helsinki metropolitan area, Finland, year 2008 (*N* = 158)

Characteristics	*N* (%)
Sex	
Male	116 (73.4%)
Female	42 (26.2%)
Marital status	
Single	75 (47.5%)
Divorced	61 (38.6%)
Married	13 (8.2%)
Widowed	4 (2.5%)
Unknown	5 (3.1%)
Length of homelessness	
< 1 year	60 (38.0%)
1–5 years	50 (31.6%)
> 5 years	37 (23.4%)
Unknown	11 (7.0%)
Reasons for evictions and homelessness	
Disturbing lifestyle	49 (31.0%)
Unpaid rent	30 (19.0%
Divorce or separation	12 (7.6%)
Termination of temporary tenancy agreement	12 (7.6%)
Voluntary termination of contract	8 (5.1%)
Unknown	47 (29.7%)
Income	
Pensions or sick leave	71 (44.9%)
Social assistance or no income	45 (28.5%)
Earnings related income allowance	24 (15.2%)
Salaries/earned income	4 (2.5%)
Unknown	14 (8.9%)
Detox treatments in the past 3 years	
0	97 (61.4%)
1–3	33 (20.9%)
4–37	28 (17.7%)
Charlson comorbidity index score	
0	113 (71.5%)
1	32 (20.3%)
2–3	8 (5.1%)
4–6	5 (3.2%)

### Primary health care visits

**Fig. 1:**
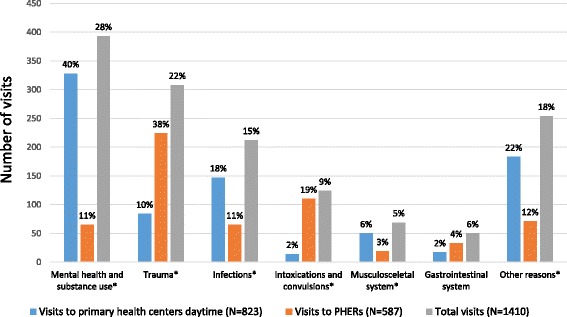
Visits to primary healthcare centers and primary health care emergency rooms (PHERs) by main reasons for visits made by homeless persons in Helsinki metropolitan area, Finland, year 2008 (*N* = 158) during 3 years. The percentages shown are percentages of visits to primary healthcare centers, PHERs and of the total amount of visits respectively. In the categories marked with a * there is a statistically significant difference between the number of visits to primary health care centers day time and PHERs for the main reason in question

During the 3 years prior to the assessment night in a shelter the 158 homeless persons in the cohort had made altogether 1410 visits to a physician in primary health care (Fig. 1). Out of these visits 546 took place in the last year prior to the assessment (mean 3.5 visits/persons during the last year). Out of all visits analyzed 58.4% took place during daytime to primary health care centers and 41.6% after hours to PHERs.

The three most common reasons for daytime visits to primary health care centers were 1) visits related to mental health and substance use (39.9%), 2) infections (17.9%), and 3) trauma (10.2%), and the three most common reasons for after hours visits to PHERs were 1) trauma (38.2%), 2) visits due to intoxications and convulsions (18.7%), and 3) visits due to infections as well as visits related to mental health and substance use (11.1% visits respectively) (Fig. 1).

### Mental disorders and somatic comorbidity

The cohort exhibited high rates of mental disorders, with 141 persons (89.2%) having a diagnosis from the group of mental and behavioural disorders (Table 2). SUDs were dominating with a 3-year prevalence of 81.6%. The majority of study subjects used only alcohol and 15.1% used illegal drugs. The most commonly mentioned illegal drugs used in this cohort were buprenorphine and amphetamines, in 14 cases respectively (data not shown), both of which are known to be commonly used in Finland [39]. Not in a single case was heroine, crack or cocaine mentioned, and in six cases the drug problem was not specified at all or only cannabis was mentioned. Based on the prevalence of mental disorders we formed four mutually exclusive groups and found that 10.7% of the sample had no mental disorder, 50.5% had only SUDs, 7.6% had other mental disorder than SUDs, and 31.0% had dual diagnosis.

**Table 2:** Prevalence of mental disorders among homeless shelter users in Helsinki metropolitan area, Finland, year 2008 (*N* = 158)

Diagnosis	ICD-10 codes	*N* (%)
Any mental disorder	F00-F99	141(89.2%)
SUD	F10-F19	129 (81.6%)
Alcohol use only		93 (58.9%)
Illegal drugs		24 (15.2%)
Alcohol use combined with benzodiazepines		12 (7.6%)
Mood and anxiety disorders	F30-F49	31 (19.6%)
Psychotic disorders	F20-F29	19 (12.0%)
Personality disorders	F60-F69	15 (9.5%)
Organic and behavioural disorders	F00-F09 and F90-F99	7 (4.4%)
Exclusive groups used in the analyses		
SUDs without other mental disorder		80 (50.6%)
Mental disorder without SUDs		12 (7.6%)
Dual diagnosis		49 (31.0%)
No mental disorder		17 (10.7%)

We used Charlson comorbidity index to assess somatic comorbidity. The sample’s index score ranged between 0 and 6, and 28.5% had a score of 1 or more. Mild liver disease was the most commonly presenting comorbidity, due to high rates of hepatitis C and B. Chronic lower respiratory diseases (including asthma and chronic obstructive pulmonary disease) were the second most commonly occurring chronic disease.

### Associations between mental disorders and primary health care use

Table 3 shows the results from the regression analyses on the effects of mental disorders on primary health care utilization during daytime. We found dual diagnosis to be strongly associated with daytime primary health care utilization (IRR 11.0, 95% CI 5.9–20.6) when compared with those without any mental disorder. The association was somewhat weaker for those with only SUDs (IRR 4.9, 95% CI 2.5–9.9) or with other mental disorder than SUDs (IRR 5.0, 95% CI 2.4–10.8). When adjusting for age and gender (Model 2) the association between mental disorders and daytime visits to primary health care was further strengthened for all groups, giving those with dual diagnosis an IRR of 12.4 (95% CI 6.6–23.4) when compared to those without any mental disorder. In Model 3 when controlling additionally for somatic comorbidity using Charlson comorbidity index the associations were slightly attenuated in all groups, for those with dual diagnosis from 12.4 to 10.4 (95% CI 5.6–19.3) but the relationship still remained strong.

**Table 3:** Associations of mental disorders with daytime visits to primary health care, negative binominal regression analysis. Homeless persons staying in shelters in Helsinki metropolitan area, Finland, year 2008 (*N =* 158)

	Model 1		Model 2		Model 3	
	IRR	95% CI	IRR	95% CI	IRR	95% CI
No mental disorder (ref.)	1		1		1	
SUDs without other mental disorder	4.9	2.5–9.9	5.7	2.7–11.9	4.5	2.2–9.0
Mental disorder without SUDs	5.0	2.4–10.8	5.2	2.5–11.0	5.0	2.5–10.0
Dual diagnosis	11.0	5.9–20.6	12.4	6.6–23.4	10.4	5.6–19.3

ref. = reference group

Model 1: Crude model (Pseudo R2 = 0.04)

Model 2: Adjusted for age and gender (Pseudo R2 = 0.04)

Model 3: Adjusted for age, gender, and Charlson Comorbidity Index (Pseudo R2 = 0.06)

When focusing upon the after hours visits to PHERs (Table 4) we observed that both dual diagnosis and SUDs without other mental disorder were strongly associated with utilization of PHERs (IRR for those with dual diagnosis 14.1, 95% CI 6.3–31.2, and IRR for those with SUDs only 11.5, 95% CI 5.7–23.3) compared with those without any mental disorder. The association between visits to PHERs and mental disorders other than SUDs was clearly weaker (IRR 2.6, 95% CI 1.1–6.2) than for those with dual diagnosis and SUDs. When adjusting for age and gender the associations were attenuated, for those with dual diagnosis the IRR decreased from 14.1 to 11.7 (95% CI 5.3–25.9) and for persons with SUDs the IRR decreased from 11.5 to 9.5 (95% CI 4.6–19.5). Additionally, controlling for somatic comorbidity using Charlson comorbidity index had only marginal effect on the IRRs in all groups. For persons with dual diagnosis the association to primary health care use was strong for both daytime and after hours visits, for persons with SUDs only the association to primary health care use was stronger for after hours visits to PHERs than for daytime visits, and for persons with mental disorders other than SUD the association was stronger for daytime visits than for after hours visits to PHERs.

**Table 4:** Associations of mental disorders with after hours visits to PHERs, negative binominal regression analysis. Homeless persons staying in shelters in Helsinki metropolitan area, Finland, year 2008 (*N *= 158)

	Model 1		Model 2		Model 3	
	IRR	95% CI	IRR	95% CI	IRR	95% CI
No mental disorder (ref.)	1		1		1	
SUDs without other mental disorder	11.5	5.7–23.3	9.5	4.6–19.5	9.1	4.4–18.8
Mental disorder without SUDs	2.6	1.1–6.2	2.6	1.0–6.5	2.6	1.0–6.4
Dual diagnosis	14.1	6.3–31.2	11.7	5.3–25.9	11.5	5.1–25.6

ref. = reference group

Model 1: Crude model (Pseudo R2 = 0.05)

Model 2: Adjusted for age and gender (Pseudo R2 = 0.06)

Model 3: Adjusted for age, gender, and Charlson Comorbidity Index (Pseudo R2 = 0.06)

## Discussion

We analyzed the use of primary health care services among homeless shelter users in Finland and found that mental disorders were strongly associated with primary health care daytime visits and after hours visits to PHERs. To our knowledge there are no previous studies on associations between primary health care use and mental disorders among homeless people in settings with universal health care. US based studies have shown that homeless persons with mental disorders have more ambulatory visits and ED visits than homeless persons with no mental disorder [12, 33], and that especially ED visits were common among homeless persons with co-occurring SUDs and other mental illnesses [33]. Our findings suggest that in systems with universal health care the homeless population’s daytime visits to primary health care are strongly associated with mental disorders and especially with dual diagnosis.

The prevalence of SUDs in our cohort was high compared with previous studies. For example, a Swedish cohort study of homeless persons using shelters and social services for homeless [10] that used hospital discharge registers to define prevalence of mental disorders found that 42.0% of the men in their cohort had SUDs (follow-up period 1996–2002), while our cohort with morbidity data based on primary health care records had a SUD prevalence of 81.2% in the 3 year follow-up period. A large Danish cohort study of homeless shelter users [5] that used psychiatric hospital and outpatient register data to define psychiatric morbidity found it to have a similar prevalence as in the Swedish study. The difference could be explained by differing health care data used. Using primary health care records is probably a more sensitive method of finding homeless persons with SUDs since a bigger proportion of the homeless population has contact with primary health care than with specialized care. Only severe cases of SUDs that require in-patient treatment are dealt with in specialized care, while less severe cases are treated either at primary health centers or at substance use outpatient clinics. For other mental disorders the 3-year prevalence rate in our study was in line with or lower than those presented in previous studies [6]. Hence, the high prevalence of SUDs in our study can be partly explained by the relatively sensitive method used to gather data on mental disorders used, but it is also a consequence of a fairly well developed welfare system and housing services for the mentally ill that prevent the main part of homelessness due to poverty or mental health problems alone.

We did not have a matched control group in this study, but comparing to the total amount of visits to primary health care centers in Helsinki in 2008 we observe that the homeless shelter users have more visits than the average citizen in Helsinki. The residents of Helsinki made on average 0.8 daytime visits to a physician in primary health care [40]. Thus, compared with the general population in Helsinki the homeless persons made 2.5 times the number of daytime primary health care centers visits. To PHERs the homeless persons made 6.8 times the number of visits compared with the average person living in Helsinki (1.4 visits for the homeless persons compared to 0.2 for the average person in Helsinki [40]). These findings are in line with studies from other settings with universal health systems [20, 21].

The three most common reasons for visits to primary health care during daytime were visits related to mental health and substance use (over one third of all daytime visits), infections, and trauma. As medications for mental health related problems are only in exceptional cases prescribed in PHERs, persons seeking help for SUDs and other mental disorders are more likely to visit primary health centers during daytime than PHERs after hours. Visits due to infections were the second most common reason to visit primary health care, and the most common infections were dermatological infections (more than half of all visits due to infections). These infections as well as visits due to trauma were often related to substance use, i.e. the patient typically had an skin infection caused by injecting drugs or has suffered a trauma while being intoxicated, and offer a part of the explanation to why patients with dual diagnosis and SUDs have more visits to primary health care daytime.

Previous studies have shown that ED visits among homeless persons are linked to mental health and substance use problems [31, 33] and the same association can be seen for primary health care utilization. While it is of course a logical consequence that people with diagnoses seek health care, our study provides more in depth knowledge about the relationship between mental disorders and the utilization of primary health care services daytime and PHER. Looking at the PHER visits we found that SUDs (including those with dual diagnosis) were strongly linked to the number of visits, while those homeless persons with other mental disorders than SUDs had had clearly fewer visits to PHER. The most common reasons for visiting PHERs, trauma and intoxications and convulsions, were also often indirectly caused by substance use, thus explaining our finding. This is in line with previous studies on ED use showing that a substantial proportion of ED visits made by homeless persons are directly related to SUDs [16]. Effective interventions such as detoxifications and case-management programs could reduce the number of visits made by homeless persons to PHERs [41–43]. In Finland, the municipalities organize treatment for SUDs, including both outpatient care and inpatient detoxification and rehabilitation, practically always on a voluntary basis. However, this study also shows that the treatment for addiction problems in the homeless population in Helsinki is insufficient, despite the commonly occurring substance use and dependency problems only 39% of the homeless persons had been in detoxification-treatment in the past 3 years. Considering that SUDs have also been shown to be linked to high mortality among homeless using shelters and social services for homeless [3], active treatment for SUDs and lower thresholds to treatment would be important.

Many of the visits that the homeless persons made were due to acute health problems such as trauma, infections and intoxications. When grouping the visits, we also formed a group for planned check-up visits due to hypertension or diabetes, causes that are known to be common reasons for visiting primary health centers. These types of prescheduled secondary preventive visits were very scarce among the homeless population, during 3 years only 14 prescheduled control visits for hypertension or diabetes (1.0% of all visits, included in the other reasons group in Fig. 1) were made to primary health care. Considering that 22 persons in the cohort were diagnosed as hypertensive and 8 as diabetic, both conditions that according to current guidelines should be checked yearly in the primary health care [44, 45], this low number of control visits shows that despite the relatively high number of visits to primary health care, treatment for chronic disease is insufficient among the homeless population in Helsinki region. These findings support previous study results showing that homeless persons seek help late due to barriers to health care and competing needs [25, 26]. In the analyses adjusting for somatic comorbidity had only little effect on the results which also suggests that the treatment of chronic diseases is responsible for only a small proportion of the total amount of visits.

The Finnish municipalities organize primary health care services for all their residents, though several barriers to health for mentally ill have been identified [46]. At the time of the study (years 2005–2008) a person registered as homeless was automatically appointed treatment at the primary health care center in the catchment area of his/her previous address. For acute cases people were entitled to use any health center’s services but for more chronic conditions one was referred to the health care center according to one’s address. Though officially this system offered primary health care for all, it also constituted a barrier to health for homeless persons. Especially the long-term homeless persons might not have any former address in the primary health care record system or the area could be very far away from where the person actually spent his or her days. This led to situations where homeless persons were turned away with the argument of not belonging to the catchment area of the health center. In order to improve access and offer freedom of choice the system has since been changed and a homeless person can now register at any primary health care center of his or her choice. Since 2009 there are also targeted primary health care services for homeless persons in conjunction with the main shelter, and the homeless persons can choose either this tailored service or any other primary health care center. Further research is needed to study whether these efforts to improve access to primary health care have improved the health situation for the homeless population in Helsinki. However, relying on previous studies [27, 28] and clinical experience by the research group it can be assumed that this tailored service reaches the homeless population better. Considering the high prevalence of SUDs in this Finnish homeless cohort it would also be relevant to study how the mortality and service utilization differ from other settings with lower prevalence of SUDs among the homeless.

### Strengths and limitations of the study

The cohort included all homeless persons staying in shelters in Helsinki region during the selected nights which strengthens its representativeness. Representativeness for all homeless in the country [34] may be somewhat compromised because the proportion of women was slightly higher (26.2% in the cohort compared to 18.8% in the whole country) as was the proportion of persons who were long-term homeless (55.1% in the cohort compared to 45.2% in the whole country). The proportion of under 25-year-olds was smaller in this cohort of homeless persons using shelters compared to the whole homeless population in Finland (5.7% in the cohort compared to 16.6% in the country). The smaller proportion of young persons in the study cohort is explained by the definition of homelessness in Finland where also persons residing with friends and family but without a permanent home are considered homeless, and fortunately not so many of the young homeless persons have to rely on shelter services.

Using primary health care records as a data source offers both challenges and access to more in-depth information than traditional register studies. When coding the main reason for the visits we first attempted to separate the visits related to substance use from those related to other mental disorders. However, this turned out impossible due to the frequently occurring comorbidity. For instance, the presenting disorder could be depression or anxiety problems, but the records also stated an active addiction problem during the same visit. It was also impossible to separate convulsions due to withdrawal symptoms from epileptic convulsions, hence these are presented in the same group.

In a primary health care setting, there is rarely time for in-depth psychiatric diagnosis, therefore, there are probably both false positives and false negatives among the prevalence rates. However, we also noted that professionals seem reluctant to make the diagnosis of substance use disorders, and only state alcohol dependency for severe cases. There is therefore more likely a problem of under- than overdiagnosis in the data. For example, personality disorders were mentioned in only 9% of the health records and organic and behavioural disorders in 4% of them. The true prevalence of these last two is probably higher in the sample but because diagnosis in primary care setting is difficult and of little practical relevance it is rarely done.

From the demographic background data we know that 38.0% of the study sample had been homeless for less than a year. This means that the sample was not homeless for the whole period analysed. We know from recent studies on Finnish shelter users [47] that homelessness is often a recurrent problem, where homeless persons move in and out of homelessness, with periods in temporary lodgings, independent and supported housing in between. Our data did not enable us to make analysis of how primary health care utilization for this group of homeless is related to periods of homelessness and periods of housing, and to study this would be an important focus for future research.

The retrospective setup of the study offered some challenges, especially the use of a prognostic index such as Charlson comorbidity index, and can be rightfully criticized. However, considering that the chronic health conditions included in Charlson comorbidity index were in most cases present already in the beginning of the analyzed period it is still a useful variable to assess the effect of somatic comorbidity on health care use.

The scope of this study was on the primary health care use in the homeless population and we did not have access to data from substance use outpatient clinics, specialized care, private hospitals or occupational health care. Previous studies have shown that homeless persons have more hospitalizations than the general population [1, 8, 12, 17], the same can be assumed to be true for this study cohort. Having access to data from specialized care would have given us more data on the disease burden in the cohort. But considering that the homeless persons had so many visits to primary health care and by analyzing the textual content of the health records and not only relying on coded register data we believe that most of the chronic disease burden could be retrieved using this method. Private health care providers and occupational health care records were not available, but bearing in mind the poverty and unemployment level among the homeless persons, it can be assumed that very little of the health care used by the homeless persons was produced by other healthcare providers than the municipalities.

## Conclusions

In this study we examined the utilization of primary health care services among homeless people and found that dual diagnosis was particularly strongly associated with primary health care daytime visits among homeless persons, while after hours visits to primary health care level emergency rooms were strongly associated with both dual diagnosis and SUDs. Active treatment for SUDs could reduce the amount of emergency visits made by homeless shelter users. Despite a relatively high number of visits to primary health care, treatment for chronic disease such as diabetes and hypertension is insufficient among homeless persons in Helsinki metropolitan area.

## Abbreviations

CI: Confidence interval
ED: Emergency department
HIV: Human immunodeficiency virus
HUS: Hospital district of Helsinki and Uusimaa
ICD-10: International Classification of Diseases, 10th Revision
IRR: Incidence rate ratio
PHER: Primary health care level emergency room
SUDs: Substance use disorders
